# Assessment of lung toxicity caused by bleomycin and amiodarone by Tc-99m HMPAO lung scintigraphy in rats

**DOI:** 10.1007/s12149-013-0722-8

**Published:** 2013-04-05

**Authors:** G. Gumuser, K. Vural, T. Varol, Y. Parlak, I. Tuglu, G. Topal, E. Sayit

**Affiliations:** 1 Department of Nuclear Medicine, School of Medicine, Celal Bayar University, Uncubozkoy, 45030 Manisa, Turkey; 2 Department of Pharmacology, School of Medicine, Celal Bayar University, Manisa, Turkey; 3 Department of Anatomy, School of Medicine, Celal Bayar University, Manisa, Turkey; 4 Department of Histology, School of Medicine, Celal Bayar University, Manisa, Turkey

**Keywords:** Tc-99m HMPAO lung scintigraphy, Bleomycin, Amiodarone, Lung toxicity, Rat

## Abstract

**Aim:**

The purpose of the study was to determine the lung toxicity caused by amiodarone (AD) and bleomycin (BLM) in rats, by means of Tc-99m HMPAO lung scintigraphy.

**Methods:**

Thirty albino rats were randomly divided into five groups. After AD or BLM was dissolved with isotonic saline (SF), a 0.5 ml solution was applied to the right bronchus via a catheter. Group 1 (*n* = 5 rats) received a single dose of AD, group 2 (*n* = 5) received two doses of AD, group 3 (*n* = 9) received BLM, group 4 (*n* = 3) received hydrochloric acid (HCl), and group 5 (*n* = 8) received SF. Rats in groups 1, 2, 3 and 5 were given 37 MBq Tc-99m HMPAO from the tail vein on days 7, 14, 21 and 28, and at 4 and 24 h in group 4. Static images of 10 min duration were obtained at 30 and 60 min by a double-headed gamma camera (Infinia, GE, Tirat Hacermel, Israel) on 256 × 256 matrix. Regular regions of interests were drawn over the right lung (RL), left lung (LL) and the liver (Li), and lung/liver (L/Li) ratios were calculated. After the scintigraphic imaging procedures were completed, rats were killed. Lung tissues were evaluated on a scale of (+) to (+++++) for edema, alveolar structural integrity and infiltration by inflammatory cells.

**Results:**

Groups 2 and 3 showed statistically significant differences in RL/Li and LL/Li ratios, whereby RL/Li was higher than LL/Li (*p* < 0.05). There were no significant differences in RL/Li and LL/Li ratios in group 5 (*p* > 0.05). In histopathological examination, minimal damage or artifacts were observed in group 5. In group 4, almost all pathological findings were present in the right lung. Statistically significant (*p* < 0.01) histological differences were found when groups 1 and 5 were compared. More significant (*p* < 0.001) pathological effects were noted when groups 2 and 3 were compared to both groups 5 and 1. Injury was more prominent in the lung tissues of the control rats that were given HCl. Increased RL/Li ratios and histopathological findings were consistent.

**Conclusion:**

Tc-99m HMPAO lung scan are found to be useful in the identification of patients with lung toxicity. The simplicity of the procedure and lower radiation exposure are the advantages of Tc-99m HMPAO lung scan.

## Introduction

Technetium d, l-hexamethylpropylene amine oxime (Tc-99m HMPAO) can easily permeate the pores in the cell membrane because of its lipophilic characteristic, reach the endothelial cytoplasm and can detect early and minimal lung damage. Essentially, it is localized in the lung endothelia. Tc-99m HMPAO penetrates into the alveolar macrophage. Alveolar integrity can be impaired due to some lung diseases, systemic diseases and medications. There are several studies that showed an increase in Tc-99m HMPAO uptake of the lungs as a result of the impairment of alveolar integrity [1–5].

Lung toxicity is a frequent side effect of some medications. Bleomycin (BLM) is an antitumorigenic antibiotic derived from *Streptomyces verticillus*. The rate of lung damage in patients using BLM varies between 2 and 46 % and may be fatal in 1–2 % of the patients [6–10]. Amiodarone (AD) is a class 3 anti-arrhythmic benzofuran derivative that is used in life-threatening ventricular arrhythmia and atrial fibrillation. The incidence of AD-induced lung toxicity varies between 1 and 17 % [11–18]. One major complication limiting the use of BLM and AD is lung toxicity, which is difficult to diagnose by conventional methods [1–5].

The purpose of the present study was to create AD-induced and BLM-induced lung toxicities in rats and to confirm this toxicity by scintigraphic imaging using Tc-99m HMPAO. We believe that it is possible to non-invasively diagnose and follow the lung toxicity that may occur in patients in the early stages.

## Materials and methods

After receiving the approval of Board of Ethics for Animal Research of our hospital, we started the research with 30 healthy male Albino rats (Ege University Center for Experimental Animals, Turkey) weighing between 250 and 300 g. The rats were randomly divided into 5 groups. The rats were weighed, placed in the supine position under (intraperitoneal) anesthesia [ketamine: 75 mg/kg (Pfizer, Turkey) and xylazine: 10 mg/kg (Sigma, Aldrich, USA)]. Under a dissection microscope (Möller-Weder, Germany), a transverse skin incision was made and the subcutis was dissected until the trachea was visible. A transverse incision was made by iris scissors (Auriga, Pakistan) 2–3 rings below the thyroid cartridge and rats were intubated using an appropriate polyethylene catheter.

After dissolving in saline, 0.5 ml of medications were administered to the rats in group 1 through the catheter into the right bronchus. A 1.83 mmol single dose of AD (1 g, Sigma-Aldrich, USA) was dissolved in distilled water at 65 °C and given to the right bronchus of rats. At the end of the first week, one of the rats died, resulting in a total of 4 rats in group 1.

Group 2 comprised five rats. A double dose of AD was administered; first dose was followed by the second dose after 48 h, using the same method.

Group 3 comprised 9 rats, and they were administered single dose of BLM (15U, Fluka, Sigma-Aldrich, USA) at 7.5 U/kg.

The positive control group was group 4, consisted of 3 rats. They were administered a single dose of HCl (1 cc/kg 0.1 N HCl).

Group 5 comprised 8 rats and a single dose of isotonic saline (SF) was administered to the right bronchus of the rats [19, 20].

Tc-99m HMPAO (Ceretec, Amersham, UK) was prepared by adding 1110 MBq of freshly eluted Tc-99m pertechnetate to 5 ml of saline solution. Quality control was conducted using a thin layer chromatography method. The labeling efficiency of Tc-99m HMPAO was found to be 90 % and over.

Rats in groups 1, 2, 3 and 5 were administered 37 MBq of Tc-99m HMPAO from the tail vein, on days 7, 14, 21 and 28. Tc-99m HMPAO scans were performed using a double-headed gamma camera (Infinia, GE, Tirat Hacermel, Israel) equipped with low-energy high-resolution parallel-hole collimators. Data were acquired at 30 and 60 min in 256 × 256 matrix using a zoom factor of 1.5. A static anterior image of 10 min in duration was obtained.

Thirty-seven MBq of Tc-99m HMPAO was injected from the tail vein to the rats in group 4 and following the administration at 4 and 24 h, scintigraphic imaging was conducted for 10 min.

On the anterior scintigraphic images, regular areas of interest were marked on both of the lungs and liver and the mean values were calculated to evaluate lung toxicity. These values were used to determine right lung/liver (RL/Li) and left lung/liver (LL/Li) ratios.

On day 28, the rats that had the scintigraphic imaging were killed with intraperitoneal injection of high-dose ketamine (50 mg/kg × VA) and xylazine (5 mg/kg × VA) and their lungs were removed. The lung samples were bisected, fixed in formalin for 24 h, hydrated through a graded series of ethanol and embedded in paraffin. Five μm thick sections were deparaffinized and stained with hematoxylin–eosin (H–E). The sections were examined under a light microscope (Olympus BX-40) and photographed by an observer blind to the groups. The images were transferred onto a computer for morphometric analyses [21].

Using a blind design, sections were analyzed for edema, alveolar destruction and the infiltration by the inflammatory cells. According to this analysis, lung damage was graded as: normal or technical damage (+), congestion (++), vascular congestion and interstitial edema (+++), minimal damage in alveolar structure and minimal infiltration of inflammatory cells (++++), massive damage in alveolar structure and massive infiltration of inflammatory cells (+++++). SPSS 11 was used for statistical analysis.

For the comparison of the RL/Li and LL/Li ratios, paired *t* test was used. A statistical significance level of 0.05 was adopted.

## Results

The RL/Li, LL/Li ratios and *p* values of rats in group 1 are given in Table 1. A statistical significant difference was observed in RL/Li and LL/Li ratios of rats in group 1 taken on day 7 both at 30 and 60 min values (*p* < 0.03). Although the RL/Li ratio on day 14 was significantly higher than the LL/Li ratio at 30 and 60 min in group 1 rats, the difference was not statistically significant (*p* > 0.05). The RL/Li and LL/Li ratios on days 21 and 28 showed statistically significant differences only at 30 min (*p* < 0.004).

**Table 1:** RL/Li, LL/Li ratios and *p* values of rats in group 1

Single dose amiodarone			
	RL/Li	LL/Li	*p* value
Day 7 (5 rats)			
30 min	0.86 ± 0.06	0.75 ± 0.06	0.030*
60 min	0.82 ± 0.14	0.72 ± 0.16	0.032*
Day 14 (4 rats)			
30 min	0.84 ± 0.10	0.72 ± 0.15	0.054
60 min	0.81 ± 0.16	0.73 ± 0.19	0.140
Day 21 (4 rats)			
30 min	0.83 ± 0.09	0.73 ± 0.08	0.004*
60 min	0.83 ± 0.07	0.77 ± 0.081	0.120
Day 28 (4 rats)			
30 min	0.74 ± 0.14	0.61 ± 0.16	0.0014*
60 min	0.73 ± 0.14	0.68 ± 0.11	0.320

*RL* right lung, *LL* left lung, *Li* liver

* *p* ≤ 0.05

**Fig. 1:**
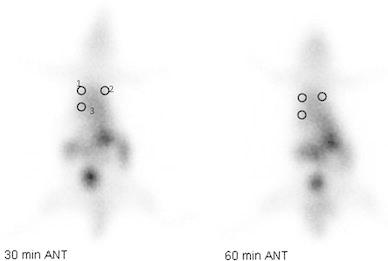
Anterior scintigraphic images of the rats that received two doses Amiodarone, obtained at 30 and 60 min using Tc-99m HMPAO 14 days after the administration and the areas of interest. *ROI 1* right lung, *ROI 2* left lung, *ROI 3* liver

The RL/Li, LL/Li ratios and *p* values of rats in group 2 are given in Table 2. In group 2, RL/Li and LL/Li ratios showed statistically significant differences on days 7 and 21, both at 30 and 60 min values (*p* < 0.05). The RL/Li and LL/Li ratios were significant at 30 min on day 14, and at 60 min on day 28 (*p* < 0.05) (Fig. 1). Although there were no significant differences for the other ratios, the RL/Li ratios were higher than the LL/Li ratios.

**Table 2:** RL/Li, LL/Li ratios and *p* values of rats in group 2

Two dose amiodarone (5 rats)			
	RL/Li	LL/Li	*p* value
Day 7			
30 min	0.87 ± 0.07	0.77 ± 0.007	0.038*
60 min	0.93 ± 0.02	0.83 ± 0.04	0.0045*
Day 14			
30 min	0.82 ± 0.18	0.76 ± 0.16	0.040*
60 min	0.88 ± 0.07	0.77 ± 0.12	0.055
Day 21			
30 min	0.78 ± 0.07	0.67 ± 0.06	0.021*
60 min	0.79 ± 0.07	0.72 ± 0.05	0.0039*
Day 28			
30 min	0.78 ± 0.07	0.71 ± 0.10	0.054
60 min	0.75 ± 0.06	0.66 ± 0.08	0.006*

*RL* right lung, *LL* left lung, *Li* liver

* *p* ≤ 0.05

**Fig. 2:**
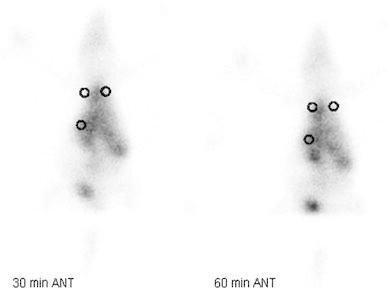
Anterior scintigraphic images of the rats that received bleomycin, obtained at 30 and 60 min using Tc-99m HMPAO 14 days after the administration and the areas of interest

The RL/Li, LL/Li ratios and *p* values of rats in group 3 are given in Table 3. Statistically significant differences were observed between the RL/Li and LL/Li ratios calculated on days 7, 14, 21 and 28, both at 30 and 60 min (*p* < 0.05) (Fig. 2).

**Table 3:** RL/Li, LL/Li ratios and *p* values of rats in group 3

Bleomycin (9 rats)			
	RL/Li	LL/Li	*p* value
Day 7			
30 min	0.66 ± 0.20	0.54 ± 0.13	0.0200*
60 min	0.62 ± 0.17	0.51 ± 0.16	0.0002*
Day 14			
30 min	0.73 ± 0.18	0.60 ± 0.16	0.0002*
60 min	0.71 ± 0.14	0.55 ± 0.10	0.0001*
Day 21			
30 min	0.71 ± 0.16	0.54 ± 0.12	0.0003*
60 min	0.78 ± 0.16	0.62 ± 0.15	0.0001*
Day 28			
30 min	0.73 ± 0.095	0.59 ± 0.08	0.0004*
60 min	0.67 ± 0.12	0.53 ± 0.008	0.0004*

*RL* right lung, *LL* left lung, *Li* liver

* *p* ≤ 0.05

**Fig. 3:**
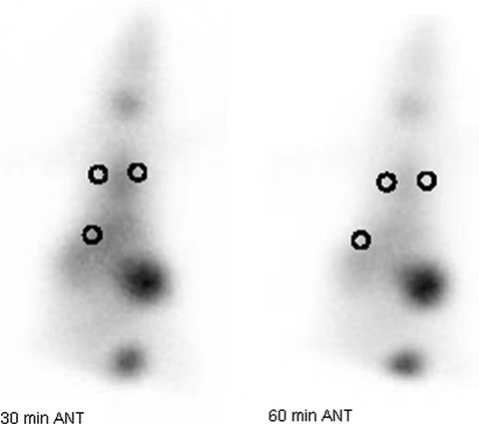
Anterior scintigraphic images of the positive control rats that received HCl, obtained at 30 and 60 min using Tc-99m HMPAO, 4 h after the administration and the areas of interest

The RL/Li, LL/Li ratios at 4 and 24 h and *p* values of rats in group 4 are given in Table 4. Statistically significant differences were observed in the RL/Li and LL/Li ratios acquired from the scintigraphic images of the three positive control rats, taken 24 h after the administration of HCl (*p* < 0.03) (Fig. 3). Although the RL/Li ratio was higher than the LL/Li ratio at 4 h, the difference was not statistically significant (*p* > 0.05).

**Table 4:** RL/Li, LL/Li ratios and *p* values of rats in group 4

Hydrochloric acid (3 rats)			
	RL/Li	LL/Li	*p* value
4 h			
30 min	0.71 ± 0.06	0.65 ± 0.09	0.09
60 min	0.76 ± 0.20	0.69 ± 0.16	0.16
24 h			
30 min	0.58 ± 0.13	0.52 ± 0.11	0.03*
60 min	0.70 ± 0.23	0.58 ± 0.20	0.02*

*RL* right lung, *LL* left lung, *Li* liver

* *p* ≤ 0.05

The RL/Li, LL/Li ratios and *p* values of rats in group 5 are given in Table 5. Isotonic saline was administered to the right lung of this group of rats and no significant difference was observed between the RL/Li and LL/Li ratios (*p* > 0.05).

**Table 5:** RL/Li, LL/Li ratios and *p* values of rats in group 5

Serum physiologic (8 rats)			
	RL/Li	LL/Li	*p* value
Day 7			
30 min	0.67 ± 0.21	0.66 ± 0.18	0.58
60 min	0.67 ± 0.18	0.68 ± 0.17	0.80
Day 14			
30 min	0.63 ± 0.18	0.64 ± 0.20	0.22
60 min	0.62 ± 0.16	0.63 ± 0.15	0.20
Day 21			
30 min	0.60 ± 0.10	0.61 ± 0.10	0.17
60 min	0.68 ± 0.14	0.68 ± 0.14	0.30
Day 28			
30 min	0.66 ± 0.15	0.66 ± 0.14	0.75
60 min	0.70 ± 0.10	0.70 ± 0.10	0.23

*RL* right lung, *LL* left lung, *Li* liver

* *p* ≤ 0.05

### Histological findings

**Fig. 4:**
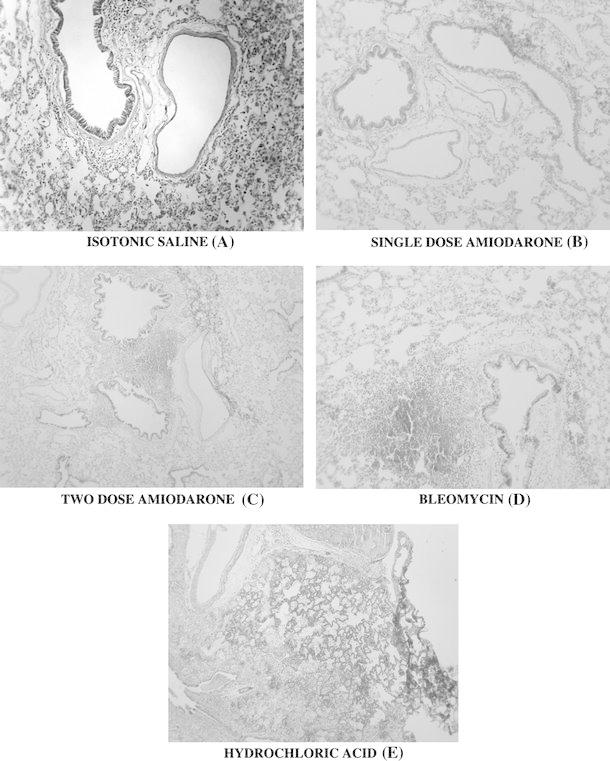
Panel of images showing the changes observed in lung tissue when saline (**a**), single-dose amiodarone (**b**), two-dose amiodarone (**c**), bleomycin (**d**) and hydrochloric acid (**e**), which served as positive control were administered. Increase in tissue edema, inflammatory cell infiltration, vacuolization within the alveoli and wall damage can be observed from **a** to **e**. HE ×200

The acute changes in the lung tissues of the subjects were examined. For histological analysis, as explained in the “Materials and methods”, the lung tissue was evaluated on the basis of interstitial edema, vascular congestion, destruction of the alveolar structure and infiltration by inflammatory cells. At low magnification (Fig. 1), group 5 showed minimal or technical damage and group 4 showed all kinds of pathological findings in the right lung. When groups 1 and 5 were compared histologically, a statistically significant difference was observed (*p* < 0.01). When groups 2 and 3 were compared with groups 5 and 1, more significant pathological changes were observed in groups 2 and 3 (*p* < 0.001) (Fig. 4). When group 2 was compared with group 3, the lung damage was similar in both groups. Administration of HCl led to more extensive lung damage than any other group (Table 4).

## Discussion

Amiodarone is a highly effective antidysrhythmic agent though it has numerous adverse effects, the most critical of which is pulmonary toxicity and consequent mortality. Processes contributing to the development of amiodarone-induced pulmonary toxicity include phospholipidosis, changes in calcium ion regulation, generation of reactive oxygen species or an amiodarone aryl radical, perturbation of cellular energy production and an immune response to the parent compound or to a metabolite [22].

With potent tumor-killing properties, bleomycins play an important role in cancer chemotherapy. Pulmonary toxicity is one of its major adverse effects. The bleomycin molecule can either intercalate into the DNA helix and separate the strands or bind iron and oxygen and form an activated complex that can release oxidants to the polynucleotide chains of DNA. Bleomycin can also cause cell damage by inducing lipid peroxidation. This may be especially important for the lung since it causes alveolar cell damage, interstitial edema, infiltration by immune cells, pulmonary inflammation and pulmonary fibrosis [6].

A wide range of radiopharmaceuticals, including Tc-99m diethylenetriaminepentaacetate (Tc-99m DTPA) [23–26], gallium 67 (Ga-67) [23, 27, 28], I-123 metaiodobenzylguanidine (MIBG) [29], I-123 iodoamphetamine (IMP), I-123 *N*, *N*, *N*-trimethyl-*N*-[2-hydroxy-3-methyl-5 iodobenzyl-1,3 propanediamine (HIPDM) and Tc-99m HMPAO [1–5, 29–33] are used to determine microvascular injuries in the lungs. I-123 MIBG, I-123 IMP, I-123 HIPDM and Tc-99m HMPAO are able to show the endothelial injury in the lungs scintigraphically [3–5]. Tc-99m HMPAO has been used in numerous studies for the imaging of chemical agent- and disease-induced lung damage [1–5, 29–33]. The simplicity of the procedure, physical characteristics, low-cost and lower radiation exposure are the advantages of Tc-99m HMPAO lung scan over Ga-67 scintigraphy and high resolution CT [28].

Various studies showed the effectiveness of HMPAO in detecting minimal endothelial lesions in the lungs. Tc-99m HMPAO is a lipophilic agent with a 380 Da molecular weight and basic and cyclic amino structure. Essentially, it is localized in the lungs in the endothelium [5, 32]. Conventional imaging methods cannot show the damage in pulmonary vascular endothelium in its early stages. Tc-99m HMPAO can penetrate through the pores in the cell membrane easily due to its lipophilic structure and can reach the endothelial cytoplasm [5]. Moreover, blood flow, presence of the alveolar macrophage and intracellular metabolism changes that may lead to a change in the glutathione help the agent to depict early and minimal lung damage [1, 5, 32].

Maintenance of the lipophilic structure and appropriate lung perfusion are the prerequisites for Tc-99m HMPAO to reach the endothelial cells. It is shown that Tc-99m HMPAO can penetrate into the broncho-alveolar liquid. It is believed that Tc-99m HMPAO uptake mostly takes place through the penetration to the macrophage [32]. Pulmonary inflammation plays an important role in pulmonary toxicity.

In the present study, as a result of the above-listed mechanisms, groups 1, 2, 3 and 4 had higher RL/Li ratios than the LL/Li ratios in the Tc-99m HMPAO lung scans. In group 5, RL/Li ratio was comparable to the LL/Li ratio. We believe that the reason why no significant difference was observed between RL/Li and LL/Li ratios in group 4 at 4th hour, despite the onset of inflammation at 4th hour, was type 2 macrophages being able to compensate for it. Necrosis and inflammation occurs at 24th hour, therefore, the scintigraphic values showed a significant difference at this time.

The RL/Li and LL/Li ratios in groups 1 and 2 did not yield a statistically significant difference; however, mean RL/Li values were higher than mean LL/Li values.

Suga et al. [5] detected early microvascular endothelial lung toxicity induced by radiation and chemical agents by Tc-99m HMPAO. Durmus-Altun et al. [23] studied AD-induced lung toxicity and administered rats AD intraperitoneally for 6 weeks and assessed lung toxicity using a Tc-99m DTPA inhalation scintigraphy. The present study differs from the study by Durmus-Altun et al. methodologically, as we have administered AD to the lungs through a catheter by opening a tracheostomy and imaging the pulmonary toxicity by Tc-99m HMPAO lung scintigraphy.

Kaya et al. [4] conducted a study investigating AD-induced lung toxicity in 3 groups of rats by Tc-99m HMPAO and they have administered AD by gavage at a dose of 10, 50 and 150 mg/kg/day. Their Tc-99m HMPAO uptake ratio was consistent with histopathological findings of AD-induced lung toxicity. In the present study, we also found results consistent with histopathological findings. Rats that were administered a single dose of AD, double dose of AD and BLM into the right lungs showed interstitial pulmonary fibrosis (IPF) in the histopathological examinations, whereas rats that were administered SF showed no pulmonary fibrosis.

Kaya et al. [34] conducted another study in which they have administered low doses of AD (5 mg/kg/day) for 6 months to New Zealand type rabbits through gavaging and again investigated lung toxicity by Tc-99m HMPAO. They observed non-specific scintigraphic and histopathological changes. The present study differs from the study by Kaya et al. methodologically as we have administered AD to the lungs through a catheter by opening a tracheostomy. Our results were also consistent with histopathological findings.

De Azambuja et al. [35] examined by Tc-99m DTPA inhalation scintigraphy 12 cases with germ cell tumors or Hodgkin lymphoma, without pulmonary disease, and had BLM as part of their chemotherapy regime. They found a significant decrease when post-treatment Tc-99m DTPA clearance half time (*t*_½_) was compared with pre-treatment values.

In the present study, we have investigated the effects of drug-induced acute lung inflammation and fibrosis. The characteristic feature of drug-induced pulmonary fibrosis is the collagen deposit in alveolar walls, which disrupts the gas exchange and limits the normal functions of the lungs [36, 37]. There are three stages of histopathological damage in the lungs: early, late and permanent. The early stage is an exudative stage and is characterized by interstitial and intra-alveolar edema, bleeding, neutrophil accumulation, fibrin plasma proteins and hyaline membranes of surfactant. At fine structure level, swollen capillaries, necrosis in the endothelium and Type 1 alveolar epithelial cells, distortion of the basal lamina are observed. In late and fibroproliferative stage, a cuboidal metaplasia that shows itself as an increase in Type 2 alveolar epithelial cells, alveolar wall thickening and fibrosis is observed. The edema starts initially in the bronchus and large vein walls and diffuse on to the alveolar wall interstitium and finally to the alveolar cavity. Reactive oxygen radicals (ROR) and proteases are usually secreted from these inflammatory cells and cause a damage in the lungs, and during the regeneration phase, an extreme fibrosis develops [38, 39].

Studies show that in drug-induced toxicities, lung damage starts in the bronchioles and at the end of the first week extend to the parenchyma. In the case of bleomycin administration, it is observed that after 15 days, inflammation decreases, collagen accumulation and cystic areas and round cells dominate. 30 days after the administration, fibrosis settles in the areas where there was inflammation. 120 days after the administration, emphysematous changes take place, alveoli expand and the walls rupture.

Macrophage inflammatory protein-1α (MIP-1α) is a protein with chemotactic properties for interstitial and alveolar macrophages. Macrophage inflammatory protein 2 (MIP-2), on the other hand, is the murine analog of interleukin-8 (IL-8) which is the neutrophil chemotactic factor in human beings. Neutrophils play an important role in the pathogenesis of IPF [40]. It is known that Type 2 cells have a stronger resistance against pathological insults. Air–blood barrier is formed by alveoli epithelia, capillary endothelia and the interstitium between them. The disruption of this barrier takes place by a change in the thickness of these three components, swelling and rupturing [21]. These early stage changes are shaped by agents called pre-inflammation cytokines such as TNF-alpha and IL-1 beta. For example, antibodies developed against these two factors may prevent the neutrophil accumulation in the lungs.

Amiodarone is an anti-arrhythmic and its most important complication is lung toxicity. Its toxicity is similar to BLM. It uses similar mechanisms as BLM. It is known that BLM induces formation of reactive oxygen metabolites such as superoxide and hydroxyl. These reactive metabolites have numerous effects such as DNA damage, lipid peroxidation, changes in the synthesis of lung prostaglandin, degradation, and increase in the synthesis of lung collagens [18, 41]. The major area of damage is Type 1 alveoli epithelial cells and pulmonary capillary endothelial cells. Type 1 pneumocyte damage is followed by Type 2 pneumocyte hyperplasia and dysplasia. Healing process in drug-induced lung damage is much slower than lung damage induced by inhalation toxins such as air pollution. It results initially in infiltration by granulocytes, lymphocytes, eosinophils and plasma cells, inflammation and cytokine release [42]. In the pathogenesis of bleomycin-induced lung disease, transforming growth factor-β (TGF-β), tumor necrosis factor-α (TNF-α), IL-1, IL-5, IL-6, platelet-derived growth factor (PDGF) and a variety of cytokines such as chemokines play important roles. In rats treated with bleomycin, TGF-β mRNA accumulates in the alveolar macrophages while anti TGF-β antibody administration minimizes the drug-induced pulmonary reactions. Similarly, BLM-sensitive IL-1 and TNF-α are produced in increasing amounts, and IL-1 receptor antagonists, anti-TNF antibodies and soluble TNF receptors alleviate the bleomycin-induced pulmonary fibrosis [42]. As a result of bleomycin-induced damage, inflammation and cytokine deregulation, fibroblasts are activated. Fibroblast and myofibroblast production increases, collagen degradation becomes inhibited leading to an increase in collagen production and fibrosis develops. As a result, cytotoxic drug-induced pulmonary damage is manifested by interstitial inflammation and fibrosis [42, 43].

In conclusion, the findings of this study showed that a significant toxicity developed especially in group 2 that received a double dose of AD and group 3 that received BLM. We believe that understanding the underlying mechanisms of this toxicity will provide valuable information for diminishing the side effects of medications. We also conclude that Tc-99m-HMPAO lung scintigraphy is a simple, sensitive and an objective method for the detection of drug-induced lung toxicity. This method also has the advantages of low-cost and low radiation exposure, when compared to Ga-67 scintigraphy and high resolution CT. Using Tc-99m HMPAO scintigraphy, we can easily detect drug-induced pulmonary toxicity caused by amiodarone or bleomycin even in the early stages, thus necessary precautions can be undertaken.
